# Development and Usability of a Novel Interactive Tablet App (PediAppRREST) to Support the Management of Pediatric Cardiac Arrest: Pilot High-Fidelity Simulation-Based Study

**DOI:** 10.2196/19070

**Published:** 2020-10-01

**Authors:** Francesco Corazza, Deborah Snijders, Marta Arpone, Valentina Stritoni, Francesco Martinolli, Marco Daverio, Maria Giulia Losi, Luca Soldi, Francesco Tesauri, Liviana Da Dalt, Silvia Bressan

**Affiliations:** 1 Division of Pediatric Emergency Medicine Department of Women’s and Children’s Health University of Padova Padova Italy; 2 Pediatric Intensive Care Unit Department of Women’s and Children’s Health University of Padova Padova Italy; 3 RE:Lab S.r.l. Reggio Emilia Italy

**Keywords:** cardiac arrest, resuscitation, mobile app, high-fidelity simulation training, cognitive aid, pediatrics, emergency medicine

## Abstract

**Background:**

Pediatric cardiac arrest (PCA), although rare, is associated with high mortality. Deviations from international management guidelines are frequent and associated with poorer outcomes. Different strategies/devices have been developed to improve the management of cardiac arrest, including cognitive aids. However, there is very limited experience on the usefulness of interactive cognitive aids in the format of an app in PCA. No app has so far been tested for its usability and effectiveness in guiding the management of PCA.

**Objective:**

To develop a new audiovisual interactive app for tablets, named PediAppRREST, to support the management of PCA and to test its usability in a high-fidelity simulation-based setting.

**Methods:**

A research team at the University of Padova (Italy) and human–machine interface designers, as well as app developers, from an Italian company (RE:Lab S.r.l.) developed the app between March and October 2019, by applying an iterative design approach (ie, design–prototyping–evaluation iterative loops). In October–November 2019, a single-center nonrandomized controlled simulation–based pilot study was conducted including 48 pediatric residents divided into teams of 3. The same nonshockable PCA scenario was managed by 11 teams with and 5 without the app. The app user’s experience and interaction patterns were documented through video recording of scenarios, debriefing sessions, and questionnaires. App usability was evaluated with the User Experience Questionnaire (UEQ) (scores range from –3 to +3 for each scale) and open-ended questions, whereas participants’ workload was measured using the NASA Raw-Task Load Index (NASA RTLX).

**Results:**

Users’ difficulties in interacting with the app during the simulations were identified using a structured framework. The app usability, in terms of mean UEQ scores, was as follows: attractiveness 1.71 (SD 1.43), perspicuity 1.75 (SD 0.88), efficiency 1.93 (SD 0.93), dependability 1.57 (SD 1.10), stimulation 1.60 (SD 1.33), and novelty 2.21 (SD 0.74). Team leaders’ perceived workload was comparable (*P*=.57) between the 2 groups; median NASA RTLX score was 67.5 (interquartile range [IQR] 65.0-81.7) for the control group and 66.7 (IQR 54.2-76.7) for the intervention group. A preliminary evaluation of the effectiveness of the app in reducing deviations from guidelines showed that median time to epinephrine administration was significantly longer in the group that used the app compared with the control group (254 seconds versus 165 seconds; *P*=.015).

**Conclusions:**

The PediAppRREST app received a good usability evaluation and did not appear to increase team leaders’ workload. Based on the feedback collected from the participants and the preliminary results of the evaluation of its effects on the management of the simulated scenario, the app has been further refined. The effectiveness of the new version of the app in reducing deviations from guidelines recommendations in the management of PCA and its impact on time to critical actions will be evaluated in an upcoming multicenter simulation-based randomized controlled trial.

## Introduction

Pediatric cardiac arrest (PCA), although rare, is an important public health issue due to its high mortality and morbidity, its complex time-dependent management and emotional burden, its social and economic costs, and differences with adult cardiac arrest (CA) [1-5].

International guidelines by relevant societies are periodically updated to help health professionals provide the best evidence-based basic and advanced care to improve the management and outcome of PCA [6-10]. Nevertheless, deviations from guideline recommendations occur frequently in the management of CA [11-19] and are associated with poorer clinical outcomes [20,21].

Different cognitive support tools have been developed and tested in order to improve adherence to guideline-recommended management of both adult CA and PCA, with variable results. Most of these tools are devices that provide real-time audiovisual feedback on the quality of chest compressions. Such tools have shown to be effective in improving the quality of compressions [22-25]. Several tools have been conceived to guide bystanders’ management of out-of-hospital CA (OHCA) through audio/video support by means of mobile phones [26]. Augmented reality glasses have also been studied to communicate with a remote intensivist to support the management of in-hospital PCA [27] or to display Pediatric Advanced Life Support (PALS) guidelines to the team leader during a PCA scenario [28] with partial benefit in improving resuscitation performance. In addition, numerous apps have been created to support the management of CA, mainly for OHCA. However, a recent systematic review outlined how the majority of these apps are not tested for content, usability, and effectiveness, even though many have already been released and are available on app stores [29].

Research on apps developed and tested to guide the management of in-hospital PCA is very limited [30], and to the best of our knowledge, no app has so far been tested for both its usability and its effectiveness in guiding the management of PCA.

Based on the deviations from guidelines recorded on a prior study conducted by our research team in PCA simulation scenarios [31], we set out to design, develop, and test a new interactive multimodal (audio–visual) cognitive aid in the format of a tablet app.

The primary aim of this pilot study was to refine the app and to test its usability and impact on team leader’s workload using high-fidelity simulation. As a secondary aim, we explored the trend in the occurrence of deviations from guidelines.

## Methods

### App Development

We designed and developed an app for tablet that we named PediAppRREST, which is the result of the collaboration between a pediatric research team, including physicians and researchers from the Pediatric Emergency Department and the Pediatric Intensive Care Unit of the University Hospital of Padova (Italy), and human–machine interface designers, human factor experts, and app/software developers of RE:Lab S.r.l., an Interaction Engineering company (Reggio Emilia, Italy).

The app was designed to guide the team leader to perform resuscitation interventions in the sequence/timing and modality reported by the American Heart Association (AHA) PALS 2015 guidelines [6-8]. In the design process we took into account the results of a prior study conducted by our research team [31], which assessed deviations from guidelines in PCA simulation scenarios managed by pediatric residents without the use of any cognitive aid.

We developed the app between March and October 2019. As a first step, the research team defined the actions to be displayed in separate screens, the flow/pathways, and the additional features that were deemed helpful to guide resuscitation and achieve a high-quality cardiopulmonary resuscitation (CPR), based on recommended PALS guidelines/algorithms. We then progressively refined and validated the cognitive aid following an iterative prototyping development approach [32]. In the development phase, serial testing of the app by research staff revealed bugs and highlighted the need for refinements concerning the information layout and organization, the user interface navigation flow, and the naming conventions. Bugs and re-design suggestions were implemented by the Interaction Engineering company.

### App Description

Directions on recommended interventions, following the order reported in the PALS algorithms, are sequentially displayed in the app which has been designed as a *checklist* app. Indeed, progression to the next screen is allowed once the recommended actions are tapped by the user, to indicate they have been read and likely performed.

**Figure 1:**
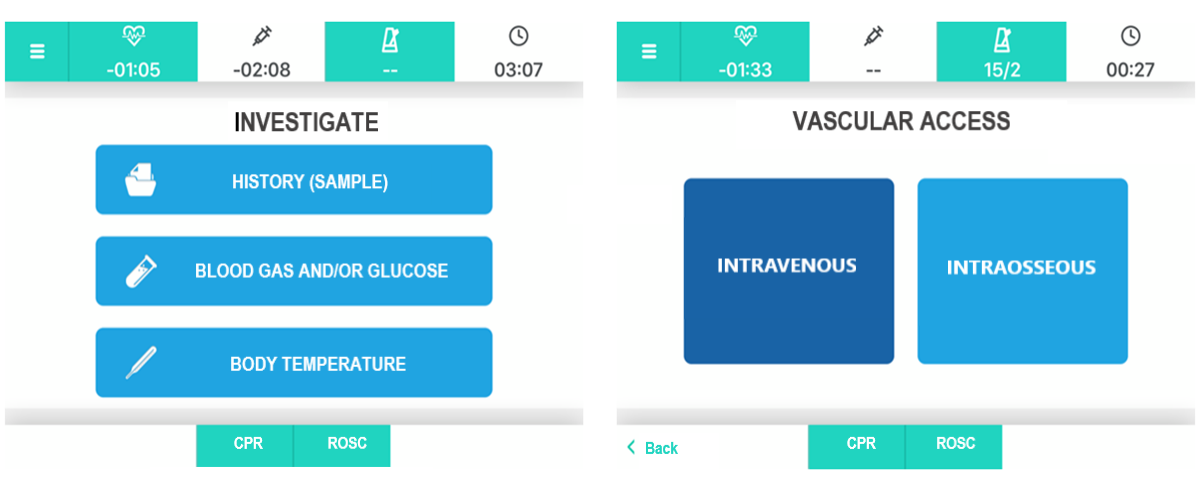
Sequential versus alternative choices. CPR: cardiopulmonary resuscitation; ROSC: return of spontaneous circulation.

The main criteria applied in the user experience design phase of the app have been (1) timely information (each screen gathers only the necessary information for each phase of the PALS algorithm, communicating it both visually and acoustically, with the aim of reducing the load on the team leader’s working memory and relying on a multichannel communication); (2) priority (actions [ie, epinephrine administration] triggered by timers have priority on other actions displayed on the screen); and (3) sequential versus alternative choices (decisions that team leaders must take into consideration concern actions and choices to be performed sequentially or alternatively). Hence, sequential actions are displayed with rectangular buttons, aligned vertically on the page, whereas alternative choices are organized with square buttons, aligned horizontally on the screen (Figure 1). The app is currently in Italian, but a multilingual version is under development (an English translation of the screen content is herein provided to ease the understanding of this article).

**Figure 2:**
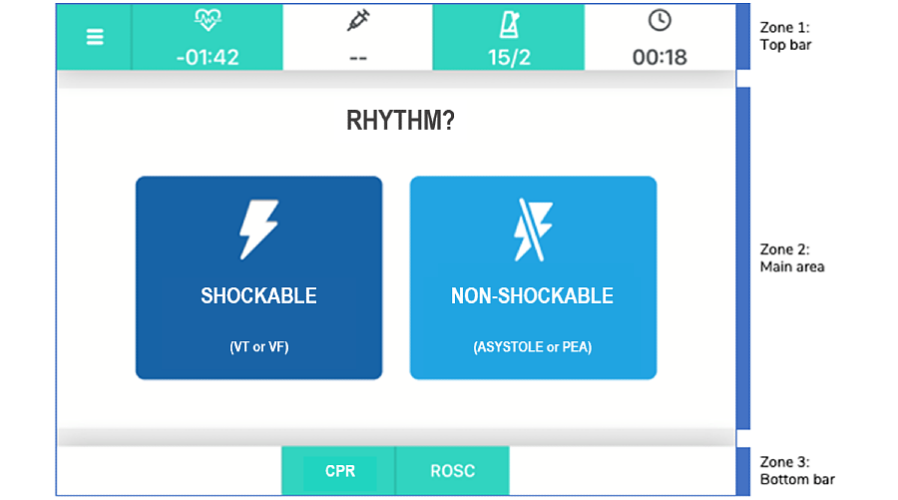
User interface main areas. CPR: cardiopulmonary resuscitation; ROSC: return of spontaneous circulation; VF: ventricular fibrillation; VT: ventricular tachycardia.

Each screen is structured into 3 zones (Figure 2): zone 1 (top bar), zone 2 (main area), and zone 3 (bottom bar).

**Figure 3:**
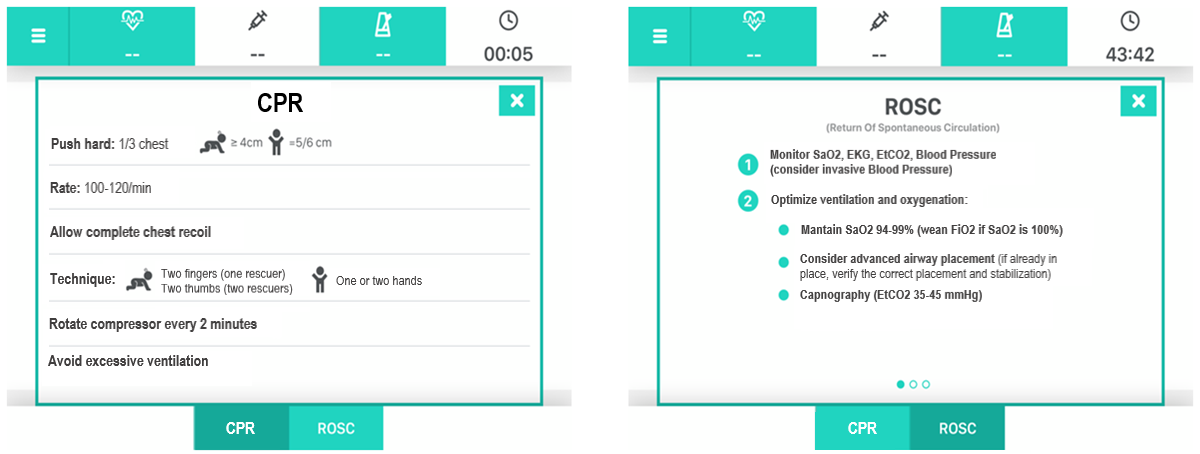
Cardiopulmonary resuscitation (CPR) and return of spontaneous circulation (ROSC) information. EKG: electrocardiogram; EtCO2: end-tidal CO2; FiO2: fraction of inspired oxygen; SaO2: oxygen saturation measured with pulse oximetry.

The main area presents the actions to be performed (with buttons of the same shape and color) or a question with different choices (buttons with different colors). Once the user taps on a button, the flow of prompts will progress following the user’s choices. On the top bar a menu-log button, a 2-minute countdown clock for repeat rhythm check button, a button with countdown for medications, a metronome button, and a total counter are displayed. The metronome button can be activated by a tap: this is a sound guide to perform compressions at the recommended rate (100-120/minute). On the bottom bar, CPR and Return of Spontaneous Circulation (ROSC) buttons are available at any time: the CPR button opens up a recap of the characteristics of a high-quality CPR, whereas the “ROSC” button summarizes the recommended management when ROSC is achieved (Figure 3).

The flow of actions that pops up in the app main area follows 2 different pathways based on the identified cardiac rhythm on the monitor (shockable versus nonshockable rhythms), as per PALS algorithms.

**Figure 4:**
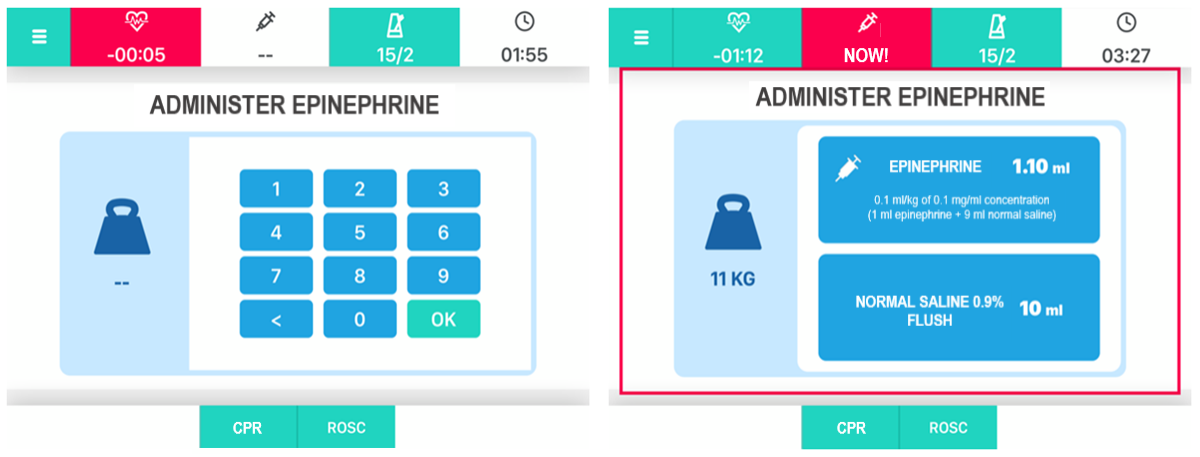
Epinephrine administration screens. CPR: cardiopulmonary resuscitation; ROSC: return of spontaneous circulation.

The app provides assistance with shock delivery, in case of a shockable rhythm, and the preparation/administration of medications, prompting the correct doses (automatically calculated on patients’ weight) and time intervals of administration (Figure 4).

Every 2 minutes the app acoustically and visually reminds the user to check the rhythm and, in case of a shockable rhythm, to deliver a shock.

**Figure 5:**
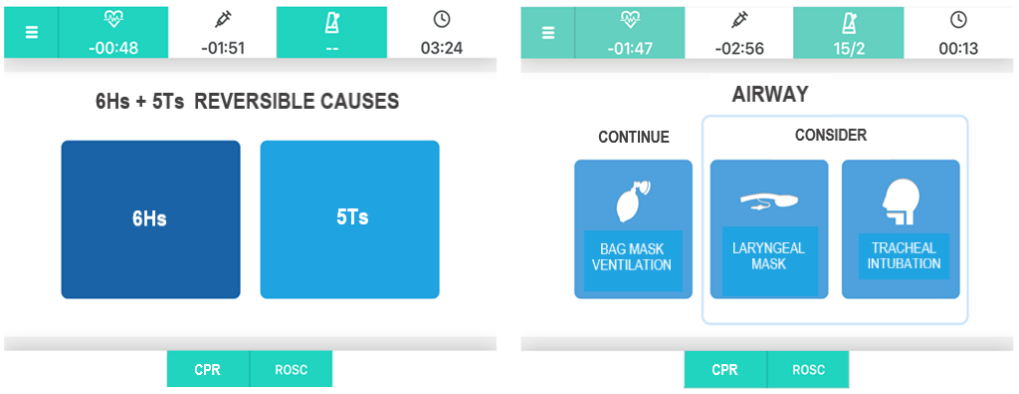
Reversible causes and airway management screens. CPR: cardiopulmonary resuscitation; ROSC: return of spontaneous circulation.

Finally, the app prompts to search/treat reversible causes of CA and to correctly manage the airway (Figure 5).

The app also gives audio prompts, suggesting the user to perform the actions shown on the main area of the screen. The user can navigate the app only using touch gestures. Voice interaction has not been integrated due to the characteristics of the resuscitation environment, which would impede accurate recognition of vocal commands.

All actions done by the user are sequentially saved on the device in the *log* function to store information that can be retrieved for any documentation purpose.

### Pilot Study

We conducted a single-center simulation-based pilot nonrandomized controlled study in October–November 2019 at the University Hospital of Padova, Italy. Although our study is not a randomized controlled trial (RCT), we followed the guidelines for reporting simulation-based studies as far as applicable [33]. All the teams of the intervention group had to manage a standard simulated PCA scenario with the use of the same version of the PediAppRREST app, while the teams of the control group managed the same scenario without the app, following usual practice. We chose a case of nonshockable PCA because asystole/pulseless electrical activity are the most frequent initial CA rhythms in children [4]. Participants were pediatric residents in their third/fourth/fifth year of their pediatric residency program who had AHA-PALS provider certification. To obtain this certification providers have to undertake a standardized resuscitation course with theorical and simulation-based education [34]. Residents unable to attend the simulations because of maternity/sick/personal leave or training abroad were excluded from the study.

Further details regarding the study methodology and procedures are described in Multimedia Appendix 1.

Written informed consent for participation was obtained from all the participants. The study was approved by the Hospital Ethics Committee as an educational project.

### Outcomes

The primary outcomes of our study were the usability of the app and the team leader’s workload. They were measured by 2 validated questionnaires, the User Experience Questionnaire (UEQ) [35-38] and the National Aeronautics and Space Administration Raw-Task Load Index (NASA RTLX) questionnaire [39,40], respectively.

Secondary outcomes were qualitative feedback on the app provided by participants, preliminary data on deviations in management from PALS guidelines recommendations, time to epinephrine administration, and resuscitation performance of the teams evaluated with the validated Clinical Performance Tool (CPT) [41,42].

### Research Measures

#### The User Experience Questionnaire

The UEQ is a validated questionnaire which comprises 26 items. Each item is represented by 2 terms with opposite meanings that the user evaluates on a 7-point Likert-type scale (from –3 to +3). The 26 items are grouped into 6 scales that cover both classical usability aspects (efficiency, perspicuity, dependability) and user experience aspects (attractiveness, stimulation, novelty). The range of each scale is also between –3 and +3. The standard interpretation of the scale is that values between –0.8 and 0.8 represent a neutral evaluation of the corresponding scale, values over 0.8 represent a positive evaluation, and values less than –0.8 a negative evaluation [35-38].

#### The NASA Raw-Task Load Index

The NASA RTLX is a simplified version of the NASA-Task Load Index which is a subjective multidimensional tool designed to assess workload. Six subscales represent different domains of the perceived workload: mental demand, physical demand, temporal demand, frustration, effort, and performance. Each domain is clearly defined and rated by participants through a 0 to 100 scale with 5-point steps. The ratings of the 6 subscales are simply averaged to create an estimate of overall workload, defined as low (<40), moderate (between 40 and 60), and high (>60) [39,40].

#### Qualitative Feedback

The qualitative feedback on user app interaction was collected through open-ended questions in the postscenario questionnaire (“What are the main difficulties you have encountered in the use of the app and/or tablet?”, “Do you have any suggestions to improve the app or its use?”) and through the postscenario debriefing. Feedback from participants was categorized by common themes.

#### Deviations From PALS Guidelines

Deviations from PALS guidelines recommendations were defined as delays and errors according to a novel checklist adapted to our intervention and scenario. We derived this new measure from the checklist, denominated c-DEV, published by Wolfe et al [20] and we integrated it with evidence-based guidelines [6-8], previously reported scoring tools [41-44], and checklists [45,46]; we named our new modified checklist c-DEVplus (Multimedia Appendix 2). It includes 16 items which represent correct critical actions for pediatric resuscitation. Each item is associated with a score as follows: 0 (action correctly and timely performed, as described in the item) or 1 point (action not undertaken or undertaken incorrectly or with wrong timing). The points of each action were summed and expressed as a total score ranging from 0 to 16. A higher c-DEVplus total score corresponds to more deviations from the guidelines. Time to epinephrine administration was measured in seconds from the recognition of CA to the moment epinephrine was administered.

#### The Clinical Performance Tool

The CPT is a performance assessment tool and a validated scoring system designed based on PALS algorithms comprising different tasks. Each task is scored as follows: not performed (0 points), performed partially, incorrectly, or late (1 point); and performed completely, correctly, and timely (2 points). Thus, the tool assesses sequence, timing, and quality of specific actions during different simulated scenarios [41,42]. In our study we used the section related to asystole and the reviewer assigned a score from 0 to 13 to each scenario.

### Statistical Analysis

The characteristics of the study participants, stratified by group allocation (control vs intervention), and the outcome variables, were summarized using descriptive statistics, and compared between the 2 groups using Mann–Whitney *U* tests for continuous variables, and chi-squared tests or Fisher exact tests for categorical variables. Data were entered into an Excel database (Microsoft) and were analyzed using Stata (version 13; StataCorp). *P*-values were two-sided, and differences were considered significant if *P* was <.05. The statistical analysis on the app usability, obtained through the UEQ, was conducted using the UEQ dedicated software, which provides descriptive statistics, and Cronbach α coefficients for each subscale, indexing their internal consistency [38].

## Results

### Characteristics of Participants

During the study period, 63 pediatric residents were assessed for eligibility, of whom 48 (16 for each one of the 3 years of residency program involved in the study) were included in the study and divided into teams of 3. Five teams managed the case following usual care (control group), whereas 11 teams (intervention group) conducted the scenario using the support of the PediAppRREST app (Multimedia Appendix 3).

Participants’ demographic characteristics, as well as training and clinical experience of resuscitation, were comparable between the 2 groups (Multimedia Appendix 4).

### Primary Outcomes

The PediAppRREST app attractiveness, perspicuity, efficiency, dependability, stimulation, and novelty were on average evaluated positively; mean UEQ scale scores were substantially above the 0.8 cutoff. UEQ scales internal consistency varied from poor (perspicuity) to acceptable (efficiency and dependability), good (novelty), and excellent (attractiveness and stimulation); see Table 1.

**Table 1:** PediAppRREST app usability.

UEQ^a^ scale	Mean (SD)	95% CI	Cronbach α coefficient
Attractiveness	1.712 (1.434)	0.865-2.559	.98
Perspicuity	1.750 (0.880)	1.230-2.270	.55
Efficiency	1.932 (0.929)	1.383-2.481	.72
Dependability	1.568 (1.102)	0.917-2.219	.76
Stimulation	1.598 (1.333)	0.811-2.386	.91
Novelty	2.205 (0.740)	1.767-2.642	.86

^a^UEQ: User Experience Questionnaire.

Team leaders’ perceived workload was comparable between the 2 groups; median NASA RTLX score was 67.5 (IQR 65.0-81.7) for the control group and 66.7 (IQR 54.2-76.7) for the intervention group (*P*=.57).

### Secondary Outcomes

Based on the qualitative feedback provided by participants, the most frequently highlighted difficulties were (1) interacting with the screens flow because information delivery was unclear about recommendation on performance of an action versus suggestion to perform an action based on the team skillset, that is, advanced airway management (n=8 team leaders); (2) information overload in the reversible causes screen, which was perceived as too dense (n=5); (3) understanding whether the selection of an icon for a recommended action had to occur at the beginning of the action or after the action was completed (ie, users did not understand whether to select the epinephrine icon at the time of preparation or administration; n=5); and (4) interacting with the app while leading the teamwork (n=4). A less frequently reported difficulty was the lack of a traditional PALS algorithm embedded within the app (n=2). Lastly, it also emerged that longer training and familiarization with the app before the simulated scenario would have been beneficial to interact more efficiently and effectively with the app.

With respect to deviations from the guidelines, the frequency of (1) incorrect compressions-to-ventilations ratio during CPR, (2) prescription of incorrect doses/dilutions of epinephrine, and (3) lack of search/treatment of reversible causes of CA (ie, hypovolemia) were higher in the control group in comparison to the intervention group; however, these differences did not reach statistical significance (Table 2). Furthermore, although the median number of deviations (c-DEVplus score) from PALS guidelines recommendations was similar between the 2 groups, a statistically significant delay (*P*=.015) in epinephrine administration was observed for the intervention group compared with the control group (Table 2). Nevertheless, the overall clinical performance of the teams, evaluated through the validated CPT, was comparable between the 2 groups (Table 2).

**Table 2:** Resuscitation performance of the teams.

Performance	Control group (N=5)	Intervention group (N=11)	*P*-value
Incorrect compressions-to-ventilation ratio, n (%)	1 (20)	0 (0)	.31
Incorrect dose or dilution of epinephrine, n (%)	1 (20)	0 (0)	.31
Lack of search and treatment of reversible causes of cardiac arrest, n (%)	2 (40)	2 (18)	.55
c-DEVplus score, median (IQR)	6 (6-7)	6 (4-7)	.27
Time (seconds) to first epinephrine administration, median (IQR)	165 (139-173)	254 (204-290)	.015
CPT^a^ scores, median (IQR)	9 (8-10)	9 (9-10)	.77

^a^CPT: Clinical Performance Tool.

## Discussion

### Principal Results

We developed a novel cognitive aid, an app for tablet, which aims to optimize the management of PCA by facilitating increased adherence to guideline recommendations. In our pilot study, the app showed a good usability profile and its use was not associated with increased team leaders’ workload. These findings are encouraging and in contrast to data on previously developed cognitive support tools which are shown to increase users’ workload [47]. Our results lay the ground to further test in an RCT the effectiveness of the PediAppRREST app which has now been refined. In fact, based on the feedback provided by participants in this pilot study, we have modified the app to improve the app–user interaction, and integrate better the use of the tool within the scenario management flow, by minimizing possible distractions related to its use, as well as possible related interference in team communication. Participants’ feedback has also guided us in better organizing the presentation of information/prompts (wording, content per screen, definition of single management steps, type of prompts) and the flow of information in the app. We have also reduced information load per screen and endeavored to facilitate the user’s understanding of the prompts presented by the app. Lastly, participants expressed the need for a longer training and testing of the app to better familiarize with the tool. This will be taken into account for the design of the RCT protocol.

**Figure 6:**
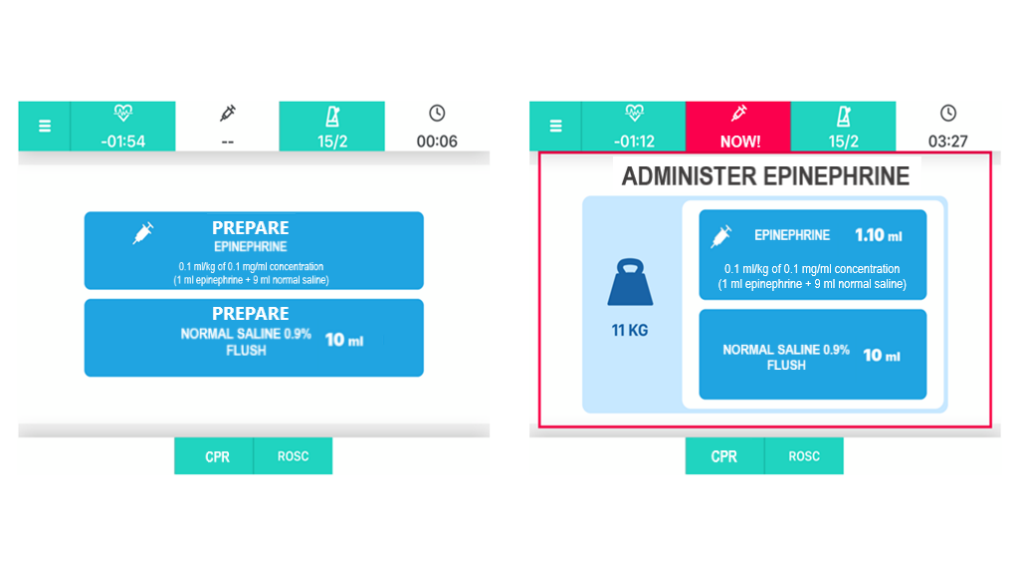
Epinephrine preparation and administration screens. CPR: cardiopulmonary resuscitation; ROSC: return of spontaneous circulation.

Our preliminary results, although based on a very limited sample size, highlighted the potential benefits, as well as the drawbacks, of using the app to guide resuscitation. Nevertheless, we are confident that the refined version of the app based on the feedback received in this pilot study and a better familiarization with it prior to its use have the potential to significantly reduce deviations from guidelines, which correlate with clinical outcomes [20], while limiting drawbacks, such as the delay in epinephrine administration. Our pilot study showed a median time to epinephrine administration approximately 90 seconds longer in the group using the app in comparison to the control group. This would be an unacceptable side effect as a recent study showed how survival decreased by 5% for every minute delay in administration of epinephrine [48]. For this reason, we have re-designed the information flow and presentation of information for the delivery of epinephrine with the aim to better reflect the management flow. Thus, we have separated the information on the preparation of epinephrine from the information on its administration (Figure 6).

We also measured team performance by means of the validated CPT score [41,42] and found similar results for the control and intervention groups. However, although this tool has been widely used in simulation research, its score has not shown to be associated with change in clinical outcomes. In addition, its items are limited and only partially reflect the potential of the app in reducing deviations from guidelines. Nevertheless, our findings are an important starting point for the design and development of an RCT to test the effectiveness of the app.

### Limitations

The results of our pilot study are preliminary and are the first step of a larger project that aims at testing the effectiveness of the PediAppRREST app. As such, they cannot be considered definitive, as the sample size is very limited. However, this pilot experience was essential to refine the newly developed tool and to verify there were the premises for a larger comparative study.

Participants of our study were exclusively pediatric residents. Although this may limit the generalizability of study findings to other clinicians, trainees are in a unique process of learning and are more used to incorporate digital assistive tools in their clinical practice. While in our setting it is easier to get trainees involved in simulation projects, than experienced clinicians, the app could be seen as a training tool in itself and future studies will be designed to test its effectiveness in knowledge and skill retention.

Our resuscitation team composition differs from other settings, where a co-team leader, a respiratory therapist, or a CPR coach is often part of the team, which includes a higher number of members. We chose a team composition that reflects the actual management at our institution during night shifts, where trainees are in the front line in the management of the first few minutes of pediatric emergencies. We felt it was important to test the device in this highly stressful and staff-limited scenario where the app could be most useful.

The intervention and control groups were unbalanced with respect to the intervention group as we needed to test the device in the first place, and a few control teams were necessary to test the overall procedures for the RCT. Similarly, although we randomly allocated residents to each team and randomly selected the teams who were performing the scenario without the app, the timing of app availability was the main determinant of our pilot study procedure and a proper randomization process will be performed for the future RCT.

A single trained reviewer rated the videos as the preliminary evaluation of team performance and deviations from guidelines were a secondary aim of this study. Two independent and trained reviewers will be ensured for the RCT, and interrater reliability will be reported and monitored. Blinding of participants and research staff was not possible because of the nature of simulation-based study. Blinding of video reviewer was not applied as video recording of the team leader using the app and the tablet was necessary to detect possible difficulties with its use. Blinding of the statistician performing data analysis will be ensured for the RCT.

The high-fidelity simulation setting during the last decades has established itself as a way to investigate rare but high-risk medical conditions. Although it does not provide data on actual patient outcomes, it is the best available way to reproduce and study rare high-stake emergencies and test novel devices developed to improve their management without compromising patients’ safety.

### Comparison With Prior Work

Several researchers have tried to create and test software products and apps to improve the quality of resuscitation. Different products have been conceived, mostly dealing with OHCA and in-hospital CA (IHCA) in adults. For instance, to help lay rescuers to manage adult cases of OHCA, different tools have been developed, such as the M-AID (an app for mobile phones [49]); a handheld personal computer software (personal digital assistant) [50]; and a voice activated decision support system, which is installed on a smartphone [51]. Conversely, other products have been developed and tested to aid professionals in training or qualified health professionals in the management of adult IHCA. These instruments comprise mobile apps, such as the iResus app developed by the Resuscitation Council UK [52] and the Medical Assistance eXpert (MAX) smartphone app [53,54], as well as an iPod Touch software (decision support) [55]. Very variable results were obtained when these tools were tested in simulation-based RCTs with only partial benefit observed in those studies that achieved positive results [50-52,54,55].

With regard to PCA, a mobile app was developed to help adolescent lay bystanders to manage an infant OHCA scenario, but an RCT showed that the participants who used the app only partially improved their performance [56]. Siebert et al [28] adapted PALS guidelines to augmented reality glasses and tested the novel cognitive aid through a simulation-based RCT on a case of shockable pediatric IHCA. The trial did not show a significant difference in time to defibrillation when using the augmented reality glasses compared with the PALS pocket reference card. However, the intervention group showed a reduction in the number of errors in defibrillation doses.

An app to help nurses prepare and administer drugs for infusion during in-hospital pediatric resuscitation has been recently developed and tested in a simulation-based RCT. The app was effective in reducing errors and time to preparation/delivery of medications compared with conventional methods [57]. Another simulation-based RCT, from the same research group, has shown a reduction in the time to critical actions and in the deviations from guidelines recommendations in the management of a shockable PCA in the group guided by an app for tablet compared with the group that used the PALS pocket reference card [30]. However, the app had not been previously tested for its usability and the sample size was limited (13 residents per group playing the team leader role, whereas the other team members were part of the research staff) [30].

To our knowledge, no app similar to the PediAppRREST has been tested in a pilot study to be refined, and to evaluate its usability and related workload before being tested in an adequately powered RCT.

### Conclusion

We developed and refined a novel interactive tablet app (PediAppRREST) for the management of PCA that has potential to reduce deviations from guidelines recommendations. The app showed a good usability profile and was not associated with higher team leaders’ workload. After this pilot testing its effectiveness will be evaluated in an adequately powered simulation-based RCT.

## Appendix

Multimedia Appendix 1 Pilot study methodology.

Multimedia Appendix 2 c-DEVplus score calculation grid.

Multimedia Appendix 3 Flowchart of participant recruitment and study group allocation.

Multimedia Appendix 4 Characteristics of participants: demographics, training and clinical experience on resuscitation.

## Abbreviations

AHA: American Heart Association
CA: cardiac arrest
CPT: Clinical Performance Tool
CPR: cardiopulmonary resuscitation
IHCA: in-hospital cardiac arrest
NASA: National Aeronautics and Space Administration
OHCA: out-of-hospital cardiac arrest
PALS: Pediatric Advanced Life Support
PCA: pediatric cardiac arrest
RCT: randomized controlled trial
ROSC: Return of Spontaneous Circulation
RTLX: Raw Task Load Index
UEQ: Usability Experience Questionnaire
