# Supplementary material for: Development and Usability of a Novel Interactive Tablet App (PediAppRREST) to Support the Management of Pediatric Cardiac Arrest: Pilot High-Fidelity Simulation-Based Study
Source: JMIR Mhealth Uhealth. 2020 Oct 1;8(10):e19070. doi: 10.2196/19070 (PMC7563631; doi:10.2196/19070)
Supplement: Multimedia Appendix 1 [file mhealth_v8i10e19070_app1.docx]

**Multimedia Appendix 1**

Development and Usability of a Novel Interactive Tablet App (PediAppRREST) to Support the Management of Pediatric Cardiac Arrest: Pilot High-Fidelity Simulation-Based Study

Corazza F*, Snijders D, Arpone M, Stritoni V, Martinolli F, Daverio M, Losi MG, Soldi L, Tesauri F, Da Dalt L, Bressan S.

## **Pilot study methodology**

### Study procedures

Participants were randomly divided in teams of three by a statistician who stratified team members by year of residency program, so that each team included a resident of the third, fourth and fifth year. This to balance the training and clinical experience of the members of each team. The teams were allocated to an intervention group and a control group following a 2:1 ratio. The first five teams who participated to the first simulation session managed the scenario without the App, as the App prototype to be used for this pilot study was still under refinement. These five teams were randomly selected out of the 16 teams included in the study. The 11 teams who participated to the following simulation sessions managed the scenario with the App. Blinding was not possible because of the nature of the intervention.

Before each simulation scenario participants completed a survey including demographic data, as well as questions on training and previous experience on resuscitation and simulation. In addition, they attended a standardized orientation briefing to the study procedures and to the simulation setting. Each team knew whether they were allocated to the intervention or control group just before the scenario. Participants in each team were asked to keep all the study procedures and scenario confidential, as disclosure of this information could have biased the results of the study. For the intervention group, team leaders underwent an 8-minute training on the App just before the scenario.

The setting, the equipment set up, the scenario, and the manikin (Gaumard HAL® S 3005) were exactly the same for both groups. Each scenario (see details below) was recorded by two cameras, placed at opposite sides of the room and capturing views of the team, the manikin, the vital sign monitor and the tablet. Each scenario lasted for 10 minutes. At end of the scenario, participants were asked to complete a usability and a workload questionnaire. Next, a 15-minute debriefing was carried out to identify and discuss deviations from recommended management as well as feedback on the App, for the intervention group.

The videos of the scenarios were reviewed, and usability issues, interventions performed, time to interventions, and deviations from guidelines were recorded by a trained reviewer.

### Simulation scenario

Case scenario: a case of non-shockable pediatric cardiac arrest caused by hypovolemia and hypoglycemia.

Team: Three pediatric residents and one confederate nurse.

Setting: off-site, in a room set reproducing the environment and the equipment of a pediatric emergency department shock room.

Introduction: “A 4-year-old child (22 kg weight) is brought to the Emergency Department by his mother; she refers diarrhea and vomiting in the past 5 days. Today the boy has been lethargic and difficult to wake up; the triage nurse evaluates the child as unconscious and brings him straight to the shock room.”

History information:

- Sign/Symptoms: vomiting and diarrhea for 5 days, reduced oral intake for the past 2-3 days;
- Allergies: no allergies;
- Medications: no medications;
- Past history: previously healthy child;
- Last meal: more than 48 hours before;
- Events: he has been sleeping for the last 2 hours, not responding to physical stimulation.

Presentation to participants: the information was provided to participants through a video where an actress plays the mother’s role.
