# Supplementary material for: Development and Usability of a Novel Interactive Tablet App (PediAppRREST) to Support the Management of Pediatric Cardiac Arrest: Pilot High-Fidelity Simulation-Based Study
Source: JMIR Mhealth Uhealth. 2020 Oct 1;8(10):e19070. doi: 10.2196/19070 (PMC7563631; doi:10.2196/19070)
Supplement: Multimedia Appendix 2 [file mhealth_v8i10e19070_app2.docx]

**Multimedia Appendix 2**

Development and Usability of a Novel Interactive Tablet App (PediAppRREST) to Support the Management of Pediatric Cardiac Arrest: Pilot High-Fidelity Simulation-Based Study

Corazza F*, Snijders D, Arpone M, Stritoni V, Martinolli F, Daverio M, Losi MG, Soldi L, Tesauri F, Da Dalt L, Bressan S.

**c-DEVplus score calculation grid**

| **c-DEVplus score** |
| --- |
| Instructions: this textbox reports the 16 items for the assessment of deviations from guideline. Failure to perform any of the actions listed below, exactly as reported, must be counted as one deviation. |
| 1. CPR started within 30 s from recognition of pulseless state |
| 1. CPR board/rigid surface positioned underneath the manikin within 60 s from recognition of pulseless state |
| 1. Compression/ventilation ratio 15:2 |
| 1. Help called (hospital emergency response system activated) within 60 s from recognition of pulseless state |
| 1. Compressors switched more than once during CPR |
| 1. ECG-monitoring started within 60 s from recognition of pulseless state |
| 1. IV/IO access called within 60 s from recognition of pulseless state |
| 1. First epinephrine called within 30 s from recognition of pulseless state |
| 1. First epinephrine administered at the correct dose and dilution^a^ and by the correct route (IV or IO), followed by a normal saline flush, while compressions are being performed, within 180 s (3 min) from recognition of pulseless state |
| 1. Second epinephrine called between 3 min and 5 min from the first administration of epinephrine |
| 1. Second epinephrine administered at the correct dose and dilution^a^ and by the correct route, followed by a normal saline flush, while compressions are being performed, within 5 min from the first epinephrine |
| 1. Blood gas called during cardiac arrest |
| 1. Reversible causes treated |
| 1. Return of Spontaneous Circulation (ROSC) obtained |
| 1. Shock not administered |
| 1. Medications other than adrenaline (e.g. amiodarone, lidocaine, atropine) not administered^b^ |

# Total score ranges from 0 to 16 deviations.

# Abbreviations: CPR=cardiopulmonary resuscitation; s=seconds; ECG=electrocardiogram; IV=intravenous; IO=intraosseous; min=minutes.

# Note: ^a^ Correct dose of epinephrine is defined as 0.01 mg/kg (or a deviation from the correct weight dose of less than 10%); correct dilution of epinephrine is defined as 1:10,000 (0.1 mg/ml); ^b^ Administration of medications to treat identified reversible causes is not considered in this item
