# Supplementary material for: Development and Usability of a Novel Interactive Tablet App (PediAppRREST) to Support the Management of Pediatric Cardiac Arrest: Pilot High-Fidelity Simulation-Based Study
Source: JMIR Mhealth Uhealth. 2020 Oct 1;8(10):e19070. doi: 10.2196/19070 (PMC7563631; doi:10.2196/19070)

**Multimedia Appendix 3**

Development and Usability of a Novel Interactive Tablet App (PediAppRREST) to Support the Management of Pediatric Cardiac Arrest: Pilot High-Fidelity Simulation-Based Study

Corazza F*, Snijders D, Arpone M, Stritoni V, Martinolli F, Daverio M, Losi MG, Soldi L, Tesauri F, Da Dalt L, Bressan S.

**Flow-chart of participant recruitment and study group allocation**


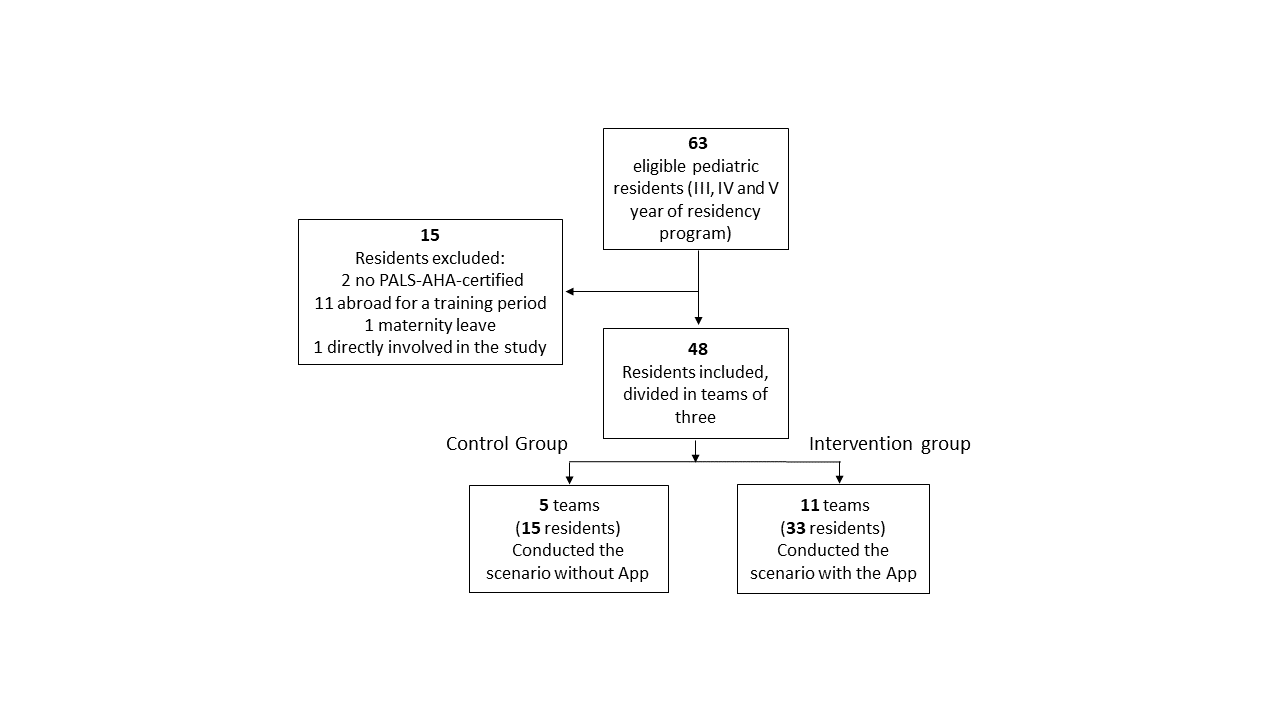

Supplement: Multimedia Appendix 3 [file mhealth_v8i10e19070_app3.docx]
