# Supplementary material for: Development and Usability of a Novel Interactive Tablet App (PediAppRREST) to Support the Management of Pediatric Cardiac Arrest: Pilot High-Fidelity Simulation-Based Study
Source: JMIR Mhealth Uhealth. 2020 Oct 1;8(10):e19070. doi: 10.2196/19070 (PMC7563631; doi:10.2196/19070)
Supplement: Multimedia Appendix 4 [file mhealth_v8i10e19070_app4.docx]

**Multimedia Appendix 4**

Development and Usability of a Novel Interactive Tablet App (PediAppRREST) to Support the Management of Pediatric Cardiac Arrest: Pilot High-Fidelity Simulation-Based Study

Corazza F*, Snijders D, Arpone M, Stritoni V, Martinolli F, Daverio M, Losi MG, Soldi L, Tesauri F, Da Dalt L, Bressan S.

**Characteristics of participants: demographics, training and clinical experience on resuscitation**

|  | | Control group  (n = 15) | Intervention group  (n = 33) | *P* value |
| --- | --- | --- | --- | --- |
| Age in years, Mdn (IQR) | | 30 (30-31) | 30 (30-31) | .88 |
| Months since PBLS certification, Mdn (IQR) | | 42 (29-53) | 42 (30-48) | .66 |
| Months since PALS certification, Mdn (IQR) | | 20 (9-28) | 20 (9-29) | .73 |
| PICU rotation previously done, n (%) | | 4 (27%) | 9 (27%) | >.99 |
| NICU rotation previously done, n (%) | | 12 (80%) | 30 (91%) | .36 |
| No PICU/NICU rotation previously done, n (%) | | 0 (0.0%) | 2 (6%) | >.99 |
| Number of simulations in the previous year, Mdn (IQR) | | 3 (0-8) | 2 (0-6) | .90 |
| **Months since the last simulation, n (%)** | |  |  | .54 |
|  | < 6 months | 8 (53%) | 14 (42%) |  |
|  | > 6 months | 7 (47%) | 19 (58%) |  |
| Months since the last simulation on CPR, Mdn (IQR) | | 9 (6-13) | 9 (6-14) | .64 |
| Number of simulations per person on CPR  in the previous year, Mdn (IQR) | | 1.5 (0-4) | 1 (0-2) | .63 |
| Participants who experienced at least  one real resuscitation in their life, n (%) | | 11 (73%) | 24 (73%) | >.99 |
| Number of real resuscitations per participant, Mdn (IQR) | | 1 (0-3) | 1 (0-3) | .84 |
| **Months since the last experience of**  **real resuscitation, n (%)** | |  |  | .72 |
|  | < 6 months | 5 (45%) | 9 (38%) |  |
|  | > 6 months | 6 (55%) | 15 (63%) |  |
| **Roles during real resuscitation scenarios, n (%)** | |  |  | .23 |
|  | Active participant | 9 (82%) | 23 (96%) |  |
|  | Observer | 2 (18%) | 1 (4%) |  |

Abbreviations: n=number; Mdn=median; IQR=interquartile range; PBLS=Pediatric Basic Life Support; PALS=Pediatric Advance Life Support; PICU=Pediatric Intensive Care Unit; NICU=Neonatal Intensive Care unit; CPR=cardio-pulmonary resuscitation.
